# Jewel in a Blouse

**Published:** 1877-11

**Authors:** 


### JEWEL IN A BLOUSE.

Passing along Sixteenth Street, some time ago, at the close of a Summer's day, we noticed a man walking before us in the commonest clothes of a laborer. He must have been over fifty years of age, and weary, too, for he walked with a weary gait, slow, and tottering. A few feet before him, near the centre of the smooth pavement, in front of Mr. Hoe's dwelling, there laid an ugly stone, not large, but just such an one as an unobservant, or old person, or little child, might stumble over. He took up the stone, carried it to the curb, laid it in the gutter, and passed along. "There's a grand heart in that poor little old man's body, in spite of its humble covering," said we to a lady with us. And it will be a lifelong regret to us that we did not overtake him and speak to him some word of cheer. That act was recognized in Heaven.

